# Early vascular healing after neXt-generation drug-eluting stent implantation in Patients with non-ST Elevation acute Coronary syndrome based on optical coherence Tomography guidance and evaluation (EXPECT): study protocol for a randomized controlled trial

**DOI:** 10.3389/fcvm.2023.1003546

**Published:** 2023-02-23

**Authors:** Yong-Xiang Zhu, Li Liang, Ramya Parasa, Zheng Li, Qian Li, Shang Chang, Wen-Rui Ma, Si-Li Feng, Yang Wang, Bo Xu, Christos V. Bourantas, Yao-Jun Zhang

**Affiliations:** ^1^Department of Cardiology, Xuzhou Third People’s Hospital, Xuzhou Medical University, Xuzhou, China; ^2^Department of Cardiology, Barts Heart Center, Barts Health NHS Trust, London, United Kingdom; ^3^Cardiovascular Devices Hub, Centre for Cardiovascular Medicine and Devices, William Harvey Research Institute, Queen Mary University of London, London, United Kingdom; ^4^Fuwai Hospital, National Center for Cardiovascular Diseases, Chinese Academy of Medical Sciences and Peking Union Medical College, Beijing, China

**Keywords:** non-ST elevation acute coronary syndrome, optical coherence tomography, percutaneous coronary intervention, early vascular healing, stent struts coverage

## Abstract

**Background:**

There is limited evidence about vessel wall healing response following implantation of next-generation drug-eluting stents (DES) in patients admitted with a non-ST elevation acute coronary syndrome (NSTE-ACS). Cumulative data indicate that optical coherence tomography (OCT) imaging can optimize percutaneous coronary intervention results and expedite stent endothelialization in the general population but there is lack of data in NSTE-ACS patients.

**Methods:**

The EXPECT study is an investigator-initiated, prospective, randomized trial to assess early vascular healing response following next-generation DES implantation in patients admitted with NSTE-ACS based on OCT guidance and evaluation. Sixty patients are randomized at 1:1:1 ratio to OCT-guided percutaneous coronary intervention (PCI) with 3-month follow-up OCT imaging (O3 group, *n* = 20), to angiography-guided PCI with 3-month follow-up OCT imaging (A3 group, *n* = 20) and to angiography-guided PCI with 6-month follow-up OCT imaging (A6 group, *n* = 20). The primary endpoint of the study is stent strut coverage rate at 3- or 6- month follow-up in the studied groups. The secondary endpoints of the study include OCT imaging endpoints, clinical endpoints, and molecular biology endpoints at the different time points. The clinical endpoints comprised of major cardiovascular adverse events and individual components. The molecular biology endpoints comprised of lipid levels and the levels of inflammatory indicators.

**Discussion:**

The findings of the EXPECT study are anticipated to provide novel insights into vessel wall healing in NSTE-ACS population following implantation of next-generation DES, underscore the value of OCT imaging in expediting strut coverage in this setting, and explore the potential of an early discontinuation of dual antiplatelet therapy (DAPT) in this population.

**Clinical Trial Registration:**

ClinicalTrials.gov, NCT04375319.

## Introduction

Over the past few decades, percutaneous coronary intervention (PCI) has significantly improved clinical outcomes in acute coronary syndrome (ACS) patients. The improved prognosis in this high-risk population has been at least partially attributed to enhanced medical therapy, the broad use of PCI as well as to the introduction of the second-generation drug-eluting stents (DES) that have increased efficacy and high safety profile (1). Current guidelines recommend treatment with dual antiplatelet therapy (DAPT) for 1 year after PCI regardless of stent types (2, 3). However, long-term DAPT may increase the risk of bleeding in the elderly, frail patients and those prone to bleeding with hematological, gastro intestinal, and liver pathologies. DAPT is also associated with a risk of serious bleeding in vulnerable patients with history of falls, previous hemorrhagic cerebrovascular events and can be a limiting factor in patients awaiting elective surgery. In the era of next-generation DES, large-scale randomized studies and meta-analyses have demonstrated that the duration of DAPT can be shortened to 6 months or even to 3 months with no clinical consequences while recent studies currently examine the safety and efficacy of aspirin single antiplatelet therapy post stent implantation (4–7).

In patients admitted with non-ST elevation acute coronary syndrome (NSTE-ACS) that have complex and highly thrombotic lesions, shorter-term DAPT can be considered only in cases of early strut coverage which constitutes a marker of early endothelialization (8). Indeed, cumulative data have underscored the prognostic implications of incomplete strut coverage indicating that this is associated with a high incidence of stent thrombosis (ST) (9) and future major adverse cardiovascular events (MACE) (10–12).

The introduction of optical coherence tomography (OCT) enabled *in vivo* assessment of strut coverage and allow us to calculate vascular repair index (13) and to identify predictors associated with a delayed vessel wall healing. In particular, stent design (i.e., stent polymer, stent strut thickness, and drug elution), strut embedment and apposition, the composition of the underlying plaque, and the time interval between stent implantation and follow-up imaging seem to determine strut coverage (14). Moreover, several studies have shown that in patients with NSTE-ACS the incidence of incomplete strut apposition is greater than in patients with a chronic coronary syndrome while the recently published study (8) has shown that the use of OCT during next-generation stent implantation is associated with a higher incidence of stent embedment and faster endothelialization comparing to angiography-guided PCI. However, most of the recruited patients in this study were suffering from a stable angina. The present investigator-initiated, prospective, randomized trial was designed to investigate the vessel wall healing response following implantation of a next-generation DES with low dose sirolimus elution in NSTE-ACS population and to explore the value of OCT imaging in expediting strut endothelialization.

## Methods and analysis

### Study design

The EXPECT study is an investigator-initiated, prospective, multicenter, randomized clinical trial (www.clinicaltrials.gov, NCT04375319). The objective of this study is to evaluate vessel wall healing response over time in NSTE-ACS patients receiving a next-generation DES implantation, and to examine the value of OCT-guided PCI in expediting stent strut coverage, aiming to underscore the potential of a lower duration DAPT in high bleeding risk population.

### Patient enrollment and randomization

Sixty patients with NSTE-ACS (includes unstable angina pectoris and non-ST segment elevation myocardial infarction) from three centers in Huaihai economic zone (Supplementary Table S1) will be enrolled in this trial. The main inclusion and exclusion criteria are listed in Tables 1, 2, respectively.

**Table 1:** Inclusion criteria.

1. Male or non-pregnant female aged 18–80 years.
2. Clinical diagnosis of acute non-ST segment elevation myocardial infarction.
3. *De novo* coronary artery lesions. Multiple target lesions should be located in different epicardial vessels.
4. Each target lesion should have a length ≤ 40 mm, and diameter between 2.5–4.5 mm by visual assessment.
5. Target lesion with ≥70% diameter stenosis or ≥ 50% diameter stenosis with myocardial ischemia evident by functional assessment.
6. Each patient is allowed to undergo a maximum of three stent implantations, if necessary (except for bailout stent implantation), each target lesion is allowed to be stented with a maximum of two stents.
7. The coronary anatomy is amenable to PCI.
8. Ability to understand the trial purpose, sign informed consent, and have good compliance with medications.

PCI, percutaneous coronary intervention.

**Table 2:** Exclusion criteria.

1. Acute ST segment elevation myocardial infarction within the past 1 month.
2. Chronic total occlusive lesion, severe left main stem stenosis, lesions with length > 40 mm, bifurcation lesion requiring double stenting, target vessel diameter > 4.5 mm, and vessels unsuitable for OCT imaging such as severely tortuous vessels and vessels with severe dissection, current infection or any active inflammatory diseases.
3. Non interpretable OCT images.
4. Severe calcific lesions that require treatment with debulking techniques.
5. In stent restenosis lesions.
6. Hemodynamic instability such as cardiogenic shock, or left ventricular ejection fraction <40% (by echocardiography or left ventriculography).
7. Renal impairment: eGFR < 60 ml/(min·1.73 m^2^) or serum creatinine>2.5 mg/dL (178 μmol/L), or patients on hemodialysis.
8. Bleeding tendency, history of active peptic ulcer, history of cerebral hemorrhage or subretinal hemorrhage, history of stroke within the past half year, or contraindications for antiplatelet or anticoagulant treatment.
9. Allergy to antithrombotic medications, contrast agent, or Cobalt Chromium.
10. Life expectancy less than 12 months.
11. Participation in other clinical trial.
12. Poor compliance, unable to provide written informed consent.
13. Heart transplantation.
14. Undergoing chemotherapy or immunosuppressive therapy.
15. Elective surgery requiring stopping anti-platelet therapy within half a year post stent implantation.
16. Platelet count lower than 100 × 10^9^/L, or more than700 × 10^9^/L, and white blood cell lower than 3 × 10^9^/L, liver diseases (such as hepatitis).

OCT, optical coherence tomography; eGFR, estimated glomerular filtration rate

The recruited patients will be randomized at 1:1:1 ratio to three groups using a computer-generated random sequence table. Patients enrolled in the first group will have OCT-guided revascularization and 3-month follow-up OCT imaging (O3 group, *n* = 20), those in the second group angiography-guided PCI and 3-month follow-up OCT imaging (A3 group, *n* = 20) while the patients recruited in the third group angiography-guided PCI and 6-month follow-up OCT imaging (A6 group, *n* = 20).

### Treatment device

All the recruited patients will be implanted with Excrossal (JW Medical Systems, Shandong, China) stent, a novel next-generation sirolimus-eluting stent with a cobalt-chromium alloy and a biodegradable polymer. The dose of sirolimus in this device is reduced to 1/3 of the former product used while the polymer consists of biodegradable polylactic acid. Following stent implantation, the polymer coating degrades into lactic acid, which expedites vascular healing process while the low dose sirolimus elution provides the advantage of accelerating endothelialization without affecting the rate of restenosis (15, 16).

### Treatment and follow-up procedures

In the OCT-guided group, OCT imaging will be performed before PCI to appropriately size stent implantation and following stenting to assess the final results and guide further optimization if needed. A final run will be performed at the end of the procedure to confirm optimal results and exclude common causes of stent failure reported in the literature (17). Patients will be given DAPT (oral aspirin 100 mg once daily, clopidogrel 75 mg once daily, or ticagrelor 90 mg twice daily) and will be put on secondary prevention medications according to the recommended ESC guidelines.

**Figure 1:**
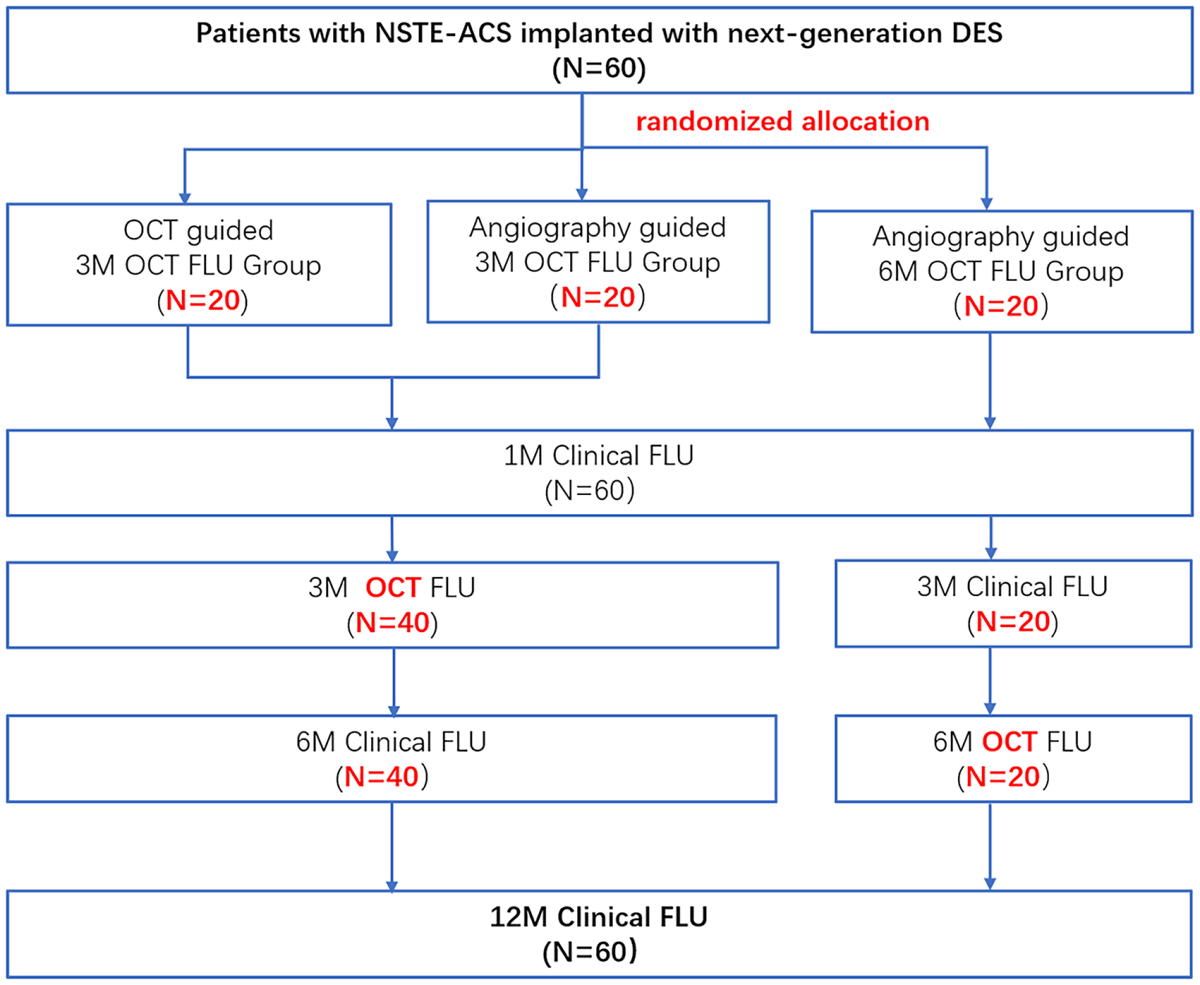
Study flow chart. NSET-ACS, non-ST elevation acute coronary syndrome; OCT, optical coherence tomography; FLU, follow up; 3 M, 3-month; 6 M, 6-month; and 12 M, 12-month.

**Figure 2:**
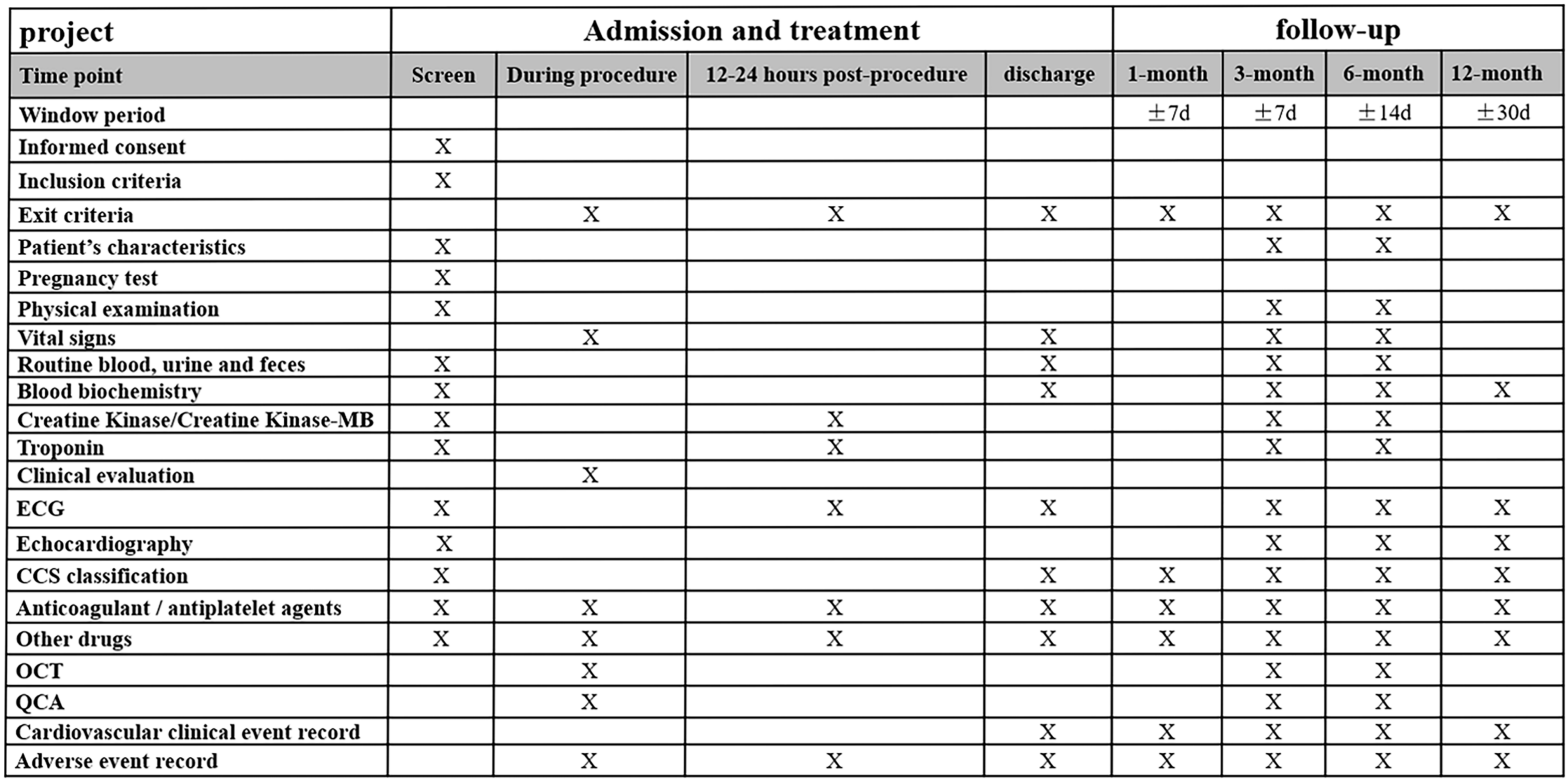
Time schedule of enrolment, interventions, and assessments of the study. ECG, electrocardiogram; CCS, Canadian cardiovascular society; OCT, optical coherence tomography; and QCA: quantitative coronary angiography.

Post discharges all patients will have a follow-up appointment or will be contacted by phone at 1 month, 3 months, 6 months, and 1 year. Subjects assigned to O3/A3 arms will undergo coronary angiography and OCT imaging at 3 months, while those assigned to A6 arm will have invasive assessment and OCT imaging at 6 months after the index procedure (Figure 1). The schedule of enrolment, interventions, and assessments is outlined in Figure 2. The data of each center will be summarized and checked by an independent data monitoring committee.

### Blood sample measurement

Blood samples (4–5 mL) will be collected from all subjects prior to the procedure and at 12–24 h after the procedure. Blood samples will be also collected at 3 and 6 months follow up. The samples will be centrifuged at 3,000 r/min for 10 min, and the serum obtained will be isolated and stored at −80°C. Serum levels of total cholesterol (TC), low-density lipoprotein (LDL-C), high-density lipoprotein (HDL-C), and triglyceride (TG) will be measured by automatic biochemical analyzer, the pentraxins 3 (PTX-3), vascular cell adhesion molecule 1 (VCAM-1), and matrix metalloproteinase 9 (MMP-9) by enzyme-linked immunosorbent assay, and the high-sensitivity C-reactive protein (hs-CRP) detected by the immune turbidity method.

### QCA and OCT data acquisition and analysis

Intracoronary nitrate will be administered prior to diagnostic angiography in all the recruited patients unless contraindicated due to low blood pressure. Antithrombotic therapy will be administered according to the local protocol prior to insertion of a guidewire in the coronary arteries.

In the angiography-guided group, stent implantation will be performed and optimized with angiography guidance according to local standard practice based on operator’s visual assessment (stent to artery ratio, 1:1). At the end of the procedure, an OCT examination will be performed for research purposes. The collected images will not be available to the operators and these images will be stored and transferred to a dedicated core-lab for image analysis.

In the OCT-guided groups, PCI will be performed under OCT guidance according ILUMIEN III: OPTIMIZE PCI algorithm. The overall procedural approach for OCT-guided stent implantation and post-implant optimization also follows this algorithm (18). In short, the stent size is chosen based on OCT measurement, and post-dilation performed under OCT guidance to achieve fully stent struts apposition and satisfactory stent area (19).

Optical coherence tomography images will be acquired in the culprit vessel and non-culprit vessels using a commercially available C7 or OPTIS imaging system (Abbott, Santa Clara, United States). The imaging catheter will be advanced at least 10 mm distally to the culprit lesion and will be pulled-back using an automated pull-back device under contrast media injection for blood clearance. A second pull-back will be performed in case of long lesions so as the catheter to cover the entire segment of interest.

Repeat coronary angiography and OCT imaging will be also performed at 3- or 6-month follow-up according to the study protocol. The angiographic and OCT imaging data collected at baseline and follow-up will be anonymized and transferred to an independent core laboratory for further analysis.

### Study endpoints

The primary endpoint of the study is the incidence of stent strut coverage (%) at 3- or 6-month follow-up.

The secondary endpoints of the study include angiographic, OCT, and clinical and biomarker outcomes that are listed in Table 3.

**Table 3:** Endpoints of the trial.

Primary endpoint
The degree of stent struts neointimal coverage (%) at 3 and 6 months
Secondary endpoint
OCT endpoint: Assessed at 3 and 6 months
1. Average and minimal stent diameter, area, and volume.
2. Average and minimal lumen diameter, area, and volume.
3. Average and minimal strut coverage thickness.
4. Strut malposition rate.
5. Vascular repair index.
Clinical endpoints: Assessed at 1, 3, 6, and 12 months
1. Major adverse cardiac events (MACE) composite of cardiac death, target vessel myocardial infarction (TV-MI), and clinical ischemia-driven revascularization.
2. Cardiac death.
3. Non-fatal MI.
4. All revascularization.
5. Target lesion revascularization.
6. Target vessel revascularization.
7. ARC-defined stent thrombosis (early, late, very-late, definite, probable, and possible stent thrombosis).
Molecular biology endpoints: Assessed at baseline, 3 and 6 months
1. Lipid levels (total cholesterol, low-density lipoprotein, high-density lipoprotein, ratio of low-density lipoprotein to high-density lipoprotein, and triglyceride).
2. Molecule indicators (hs-CRP, PTX-3, VCAM-1, and MMP-9).

ARC, academic research consortium; hs-CRP, high sensitive C-reactive protein; PTX-3, pentraxin-3; VCAM-1, vascular cell adhesion molecule-1; and MMP-9, matrix metalloprotein-9.

### Statistical considerations

The sample size of this study is based on the previously published literature and the corresponding research hypothesis. This study intends to demonstrate that the incidence of strut coverage in the OCT-guided group is higher than that of angiography-guided group at 3-month follow-up (hypothesis 1). The second hypothesis of this study is that the incidence of stent strut coverage in the OCT-guided group at 3 months is similar to the incidence of strut coverage in the angiography-guided group at 6 months (non-inferiority analysis). If the above hypothesis is confirmed then an additional analysis will be performed that aims to demonstrate that the incidence of covered struts is higher at 3 months in the OCT-guided group compared to the rate of strut coverage noted at 6 months in the angiography-guided PCI (hypothesis 3). In order to control for the type I error, the test significance levels of hypothesis 1 and 2 are set at one-sided 0.025. Since hypothesis 3 is a sequential test carried out on the basis of hypothesis 2, the test significance level is set at one-sided 0.05.

The sample size calculation is based on the assumption that each patient will receive on average 1 stents, and that each stent will have a length of 24 mm. OCT analysis will be performed at 0.4/0.6 mm interval and that 7.9 struts in each cross section will be detected. Based on these assumptions, it is estimated that 474 strut results will be obtained from each patient. We estimate that we will need 7,305 strut estimations in each group to prove with 95% power the primary endpoint of the study. Based on the above assumptions, we estimate that 16 patients should be recruited in each group. Considering a 20% drop-off rate, we estimate that 20 patients should be included in each group (Table 4).

**Table 4:** Parametric assumptions for hypothesis.

	Experimental group coverage	Control group coverage	Non-inferiority margin	Sample size (stent struts)
Hypothesis 1	93.6%	92%	Not applicable	6,781
Hypothesis 2	95%	95%	1.3%	7,305
Hypothesis 3	96.5%	95%	Not applicable	3,912

Continuous variables will be described as means and SDs if they are normally distributed or median and interquartile range in the case of asymmetric data distribution. Categorical variables will be presented as numbers and percentages. Comparisons of numerical variables are conducted with the Student’s *t* test or Mann–Whitney’ *U* test as appropriate, categorical variables and outcomes were compared by using the chi-squared test or Fisher exact test. A statistical package (SPSS 21.0) will be used for analysis. Two-tailed *p* < 0.05 will be considered statistically significant.

## Discussion

Dual antiplatelet therapy is recommended following stent implantation, but the optimal duration remains controversial in ACS patients, the current ACC/AHA and ESC guidelines recommend DAPT for 12 months after PCI (2, 3). However, prolonged DAPT is associated with worse outcomes in high bleeding risk populations. A consensus from the Academic Research Consortium for High Bleeding Risk (ARC-HBR) defined 14 and six clinical criteria as major or minor criteria, respectively. If patients meet at least one major or two minor criteria, they are considered to be at high bleeding risk (20). According to the ARC-HBR criteria, 40% patients in real-world PCI registry are at high bleeding risk (21). These patients are often excluded from or underrepresented in clinical trials about stents and antiplatelet therapy. The development of advanced stent platforms and the optimal stent deployment—even with the use of intravascular imaging guidance—is expected to enable shorter duration of DAPT in high bleeding risk ACS patients undergoing PCI. Several studies have shown that shorter-term DAPT in ACS patients implanted with next-generation DES is non-inferior to the standard or longer duration of DAPT. More specifically the “all-comer” ITALIC randomized controlled trial demonstrated that after the implantation of next-generation DES, 6-month DAPT followed by aspirin monotherapy is non inferior to 24-month DAPT in terms of all-cause mortality, myocardial infraction (MI), target vessel revascularization (TVR), stroke, and major bleeding in a low risk profile for ischemic events population (22). Similarly, the randomized OPTIMA-C trial (NCT03056118) showed that the MACE rate at 12-month follow-up was not different in patients receiving 6-month DAPT and those treated for 12-month DAPT after implantation of next-generation DES (23). The multicenter REDUCE trial enrolled 1,496 patients admitted with an ACS treated with the next-generation COMBO stent who were randomized to 3-month (*n* = 751) or 12-month (*n* = 745) DAPT. The primary endpoint was a composite of all-cause mortality, MI, ST, stroke, TVR, and bleeding and its incidence was similar in the two groups (8.2 vs. 8.4%, *p* non-inferiority<0.001) at 1- or 2-year follow-up (11.6 vs. 12.1%, *p* = 0.76) (24).

There is compelling evidence that strut coverage is a predictor of ST (25). The next-generation DES with thinner struts, safer polymer profiles and improved drug kinetics appear to enable faster endothelialization and have known to reduce the incidence of ST compared to the first generation DES (25). The OCT sub-study of the OPTIMA-C trial revealed favorable strut coverage at 6 months after next-generation DES implantation (23), similarly the FUNCOMBO trial showed almost complete strut coverage of the next-generation DES in patients who presented with STEMI (26). Moreover, a small study reported that in the biodegradable polymer Synergy stent strut coverage was 94.5% at 3 months and 96.6% at 6 months (27) whereas the TARGET All Comers study showed a similar incidence of strut coverage in biodegradable polymer Firehawk and durable polymer XIENCE stent group (99.9 ± 0.3 vs. 100 ± 0.1%, *p* = 0.26) (28). Conversely, the biodegradable polymer BuMA Supreme stent was found to be superior to the XIENCE stent in terms of strut coverage at both 1 month and 2 months in the PIONEER-II OCT trial (29). However, stent implantation in these studies was mostly performed under angiography guidance. The DETECT-OCT (NCT01752894) trial was the first that highlighted the value of OCT imaging in optimizing stent apposition and in this way accelerating strut coverage. In this trial, the percentage of uncovered struts at 3 months was lower in the OCT-guided group (7.5%) than in the angiography-guided group (9.9%; *p* = 0.009) (8). However, the DETECT-OCT trial mainly included patients suffering from stable angina and did not include the group of patients undergoing OCT at a longer follow-up period. This study was designed to provide additional insights as it aims to include NSTE-ACS patients, and include the group of patients that will undergo OCT imaging at 6 months to examine late strut coverage in patients undergoing angiography guided PCI.

Additionally, clinical studies have found that MACE after PCI not only comes from in-stent restenosis or ST in ACS patients but also due to progression of mild to moderately graded disease in the non-culprit vessels as it is a well-known factor that patients presenting with ACS often have multivessel disease (30, 31). A study involving nearly 13,000 ACS patients showed that over 80% of ischemic events occurring after 30 days were unrelated to the culprit lesion that was stented, but were rather spontaneous, or *de novo* events in a non-culprit vessel (32). Intravascular imaging research revealed that non-culprit lesions in ACS patients are often vulnerable plaques, with characteristics such as thin-cap fibroatheroma and those with heavy lipid load. The progress and rupture of these vulnerable plaques in non-culprit vessels can also result in adverse events like thrombosis after PCI (33, 34). Therefore, understanding the evolution of plaque in non-culprit vessels is of great significance to prevent future adverse cardiac events and may potentially influence decision making in the use of DAPT duration. Thus, our study innovatively performs OCT examination in both culprit and non-culprit vessels at baseline and follow-up, to explore vulnerable plaque progression in non-target vessels, measure the changes in the thickness of the fibrous cap over lipid-rich plaques, and examine changes in macrophages accumulations and in the lipid content and in the vulnerable plaques, and their transformation at various time points. Furthermore, plaque progression is usually accompanied with lipid infiltration and inflammatory response. The positive correlation of cholesterol levels, proinflammatory cytokines, and atherosclerotic evolution is well established (35). Stent implantation is associated with vessel wall injury and endothelium disruption while it is well known that strut polymer and drug elution can trigger a pro-inflammatory and hypersensitivity reaction leading to the formation of neoatherosclerotic lesions (34). There are however limited data about the association between vulnerable plaque progression in native segments and neointima proliferation and neoatherosclerotic lesion formation in segments treated with stents. The present study has been designed to provide additional insights and explore the implications of the lipid profile and systemic inflammation assessed by circulatory biomarkers on neointima characteristics and atherosclerotic disease progression in native segments.

This study has several limitations. Firstly, only three centers in Huaihai economic zone are participated in this study. Therefore, the findings of this analysis may not be relevant to other populations. Secondly, “different operators” experience may impact the procedure outcomes, which is the source of uncontrolled bias. Finally, several studies revealed that the early strut coverage of biodegradable polymer DES is not similar with other next-generation DES (36, 37), which implies the results of our study may not be simply generalized to other DES with different design concept.

### Ethics and dissemination

The study protocol has been approved by the ethics committee of Xuzhou Third People’s Hospital, Xuzhou Cancer Hospital (Xuzhou city, Jiangsu Province). The reference number is 2019-02-006-H02. A Chinese original document and an English translation of the ethical approval document are attached at Additional file 2. Written informed consent is provided by patients before enrollment.

The study results will be submitted for publication in peer-reviewed journals. Data will be disseminated and presented at scientific meetings.

### Patient and public involvement

Patients or the public were not involved in the design, or conduct, or reporting, or dissemination plans of this trial.

## Ethics statement

The studies involving human participants were reviewed and approved by Ethics committee of Xuzhou Third People’s Hospital, Xuzhou Cancer Hospital (Xuzhou city, Jiangsu Province). The patients/participants provided their written informed consent to participate in this study.

## Author contributions

Y-XZ, LL, and Y-JZ co-designed the study protocol. Y-XZ, LL, and RP co-drafted the manuscript. ZL, QL, SC, and S-LF were involved with study conduct and data acquisition. W-RM and YW provided the statistical analysis. CB, BX, and Y-JZ further aided in assessment and revision of the protocol and revised the manuscript. All authors contributed to the article and approved the submitted version.

## Funding

This investigator-initiated study is supported by JW Medical Systems (Shandong, China, grant number: N/A), 333 High-level Talent Training Program of Jiangsu Province (grant number: BAR2018275), and full-time introduction of special medical talents in 2018 of Xuzhou city (grant number: 2019-TPRC-1). These funding bodies are only providing financial support. The authors are solely responsible for the design and conduct of this study, analysis of the study data, drafting and editing of the paper, and the final content of the paper.

## Conflict of interest

The authors declare that the research was conducted in the absence of any commercial or financial relationships that could be construed as a potential conflict of interest.

The handling editor TG declared a past collaboration with the author CB.

## Publisher’s note

All claims expressed in this article are solely those of the authors and do not necessarily represent those of their affiliated organizations, or those of the publisher, the editors and the reviewers. Any product that may be evaluated in this article, or claim that may be made by its manufacturer, is not guaranteed or endorsed by the publisher.
