# Supplementary material for: Early vascular healing after neXt-generation drug-eluting stent implantation in Patients with non-ST Elevation acute Coronary syndrome based on optical coherence Tomography guidance and evaluation (EXPECT): study protocol for a randomized controlled trial
Source: Front Cardiovasc Med. 2023 Feb 23;10:1003546. doi: 10.3389/fcvm.2023.1003546 (PMC9995825; doi:10.3389/fcvm.2023.1003546)
Supplement: Supplementary file 1 [file Table_1.DOCX]

**Table S1** A list of the participating hospitals.

| Hospital Name | Address | District/County | City | Province | Grade |
| --- | --- | --- | --- | --- | --- |
| Xuzhou Third People’s Hospital (Xuzhou Cancer Hospital) | No.131  Huancheng Road | Gulou | Xuzhou | Jiangsu | 3 |
| Xuzhou Renci Hospital | No.11  Yangshan Road | Gulou | Xuzhou | Jiangsu | 3 |
| Peixian County Guotai Hospital | Western side of Nanhuan Road Railway River | Peixian | Xuzhou | Jiangsu | 2 |

1. The third grade represent the highest-grade hospital in China.
2. Professor Yao-Jun Zhang studios are established in department of cardiology of all participating hospitals.
3. District and county are at the same administrative level in China.
